# Wilms’ tumor gene 1 regulates *p63* and promotes cell proliferation in squamous cell carcinoma of the head and neck

**DOI:** 10.1186/s12885-015-1356-0

**Published:** 2015-05-01

**Authors:** Xingru Li, Sofia Ottosson, Sihan Wang, Emma Jernberg, Linda Boldrup, Xiaolian Gu, Karin Nylander, Aihong Li

**Affiliations:** 1 Department of Medical Biosciences, Clinical Chemistry, Umeå University, By 6 M, 2nd floor, Umeå, 90185 Sweden; 2 Department of Medical Biosciences, Pathology, Umeå University, By 6 M, 2nd floor, Umeå, 90185 Sweden

**Keywords:** *WT1*, *p63*, *p53*, Cell proliferation, Squamous cell carcinoma of the head and neck (SCCHN)

## Abstract

**Background:**

Wilms’ tumor gene 1 (*WT1*) can act as a suppressor or activator of tumourigenesis in different types of human malignancies. The role of *WT1* in squamous cell carcinoma of the head and neck (SCCHN) is not clear. Overexpression of *WT1* has been reported in SCCHN, suggesting a possible oncogenic role for *WT1*. In the present study we aimed at investigating the function of WT1 and its previously identified protein partners p63 and p53 in the SCCHN cell line FaDu.

**Methods:**

Silencing RNA (siRNA) technology was applied to knockdown of WT1, p63 and p53 in FaDu cells. Cell proliferation was detected using MTT assay. Chromatin immunoprecipitation (ChIP)/PCR analysis was performed to confirm the effect of WT1 on the p63 promoter. Protein co-immunoprecipitation (co-IP) was used to find protein interaction between WT1 and p53/p63. Microarray analysis was used to identify changes of gene expression in response to knockdown of either WT1 or p63. WT1 RNA level was detected using real-time quantitative PCR (RT-qPCR) in patients with SCCHN.

**Results:**

We found that WT1 and p63 promoted cell proliferation, while mutant p53 (R248L) possessed the ability to suppress cell proliferation. We reported a novel positive correlation between WT1 and p63 expression. Subsequently, *p63* was identified as a WT1 target gene. Furthermore, expression of 18 genes involved in cell proliferation, cell cycle regulation and DNA replication was significantly altered by downregulation of WT1 and p63 expression. Several known WT1 and p63 target genes were affected by WT1 knockdown. Protein interaction was demonstrated between WT1 and p53 but not between WT1 and p63. Additionally, high *WT1* mRNA levels were detected in SCCHN patient samples.

**Conclusions:**

Our findings suggest that *WT1* and *p63* act as oncogenes in SCCHN, affecting multiple genes involved in cancer cell growth.

**Electronic supplementary material:**

The online version of this article (doi:10.1186/s12885-015-1356-0) contains supplementary material, which is available to authorized users.

## Background

Squamous cell carcinoma of the head and neck (SCCHN) is the sixth most common cancer and also the most common tumor type in the head and neck region. The 5-year survival is approximately 50% and has increased only marginally during the last decades. The molecular pathogenesis of SCCHN is not yet completely understood, a fact that complicates development of new therapeutic approaches [1]. Mutations in the *p53* gene have been reported in one to two thirds of SCCHN [2]. The p53-related transcription factor, *p63*, is reported to be overexpressed in the majority of primary SCCHN tumors [3,4]. p63 expression is regulated through two distinct promoters, giving rise to two main isoforms, TAp63 and ΔNp63. TAp63 is transcribed from the external promoter which contains the transactivating domain homologous to p53, enabling it to regulate transcription of p53 target genes. ΔNp63 is transcribed from an internal promoter and acts in a dominant negative fashion with the ability to overcome the cell cycle arrest and apoptosis normally driven by p53 [5]. The main isoform overexpressed in SCCHN is ΔNp63α, a critical pro-survival protein [6,7].

Wilms’ tumor gene 1 (*WT1*) was first identified as a tumor suppressor gene in Wilms’ tumor, a childhood kidney neoplasm [8]; later findings demonstrated oncogenic properties in other malignancies including breast [9], lung [10,11], ovarian [12,13] and brain tissue [14]. WT1 was previously found to interact with p53 and p63 at protein level in baby rat kidney cells and in Saos-2, an osteosarcoma cell line [15,16]. However, the interaction has not been studied in any other cell types yet.

In SCCHN, *WT1* overexpression has been reported by Oji et al. [17] suggesting an oncogenic property. However, no functional study has been performed to investigate the role of WT1 in SCCHN tumorigenesis.

In the present study, our aims were to investigate the function of WT1 in SCCHN and to examine possible interactions between WT1 and p63/p53. A positive correlation between WT1 and p63 was found in FaDu cells, an SCCHN cell line. ChIP analysis verified WT1 binding to the *p63* promoters, designating *p63* a target gene of WT1. The functional link between WT1 and *p63* was further demonstrated by altered expression of several known p63 target genes in WT1 knockdown cells. By silencing *WT1* and *p63* RNA, SCCHN cell proliferation was decreased. WT1 and p63 were found to generate effects on cell proliferation through multiple genes involved in cell proliferation, cell cycle regulation and DNA replication.

## Methods

### Cell culture

The FaDu cell line (ATCC HTB-43), derived from hypopharyngeal squamous cell carcinoma, was used for transfection experiments. The cells were maintained in Dulbecco’s modified Eagle’s medium (Gibco, Stockholm, Sweden) containing 10% fetal bovine serum (Gibco) in 5% CO_2_ at 37°C.

### siRNA and WT1D plasmid transfection

Pooled siGENOME SMART pool of *WT1, p63* and *p53* siRNA (Dhamacon, Chicago, USA) was used for transfection. To suppress expression of *WT1*, *p63* and *p53*, FaDu cells were transiently transfected with siRNA of *WT1* (12.5 nM/well), *p63* (5 nM/well) and p53 (5 nM/well) in six well plates (3 × 10^5^ cells/well) and 96-well plates (8 × 10^3^ cells/well). Lipofectamine RNAiMAX reagent (Invitrogen, Carlsbad, CA, USA) was used for suppression of gene expression. Cells were harvested at 24, 48 or 72 hours after transfection for further analysis. To induce WT1D overexpression, pcDNA 3.1 (+) vectors (Invitrogen, Carlsbad, CA, USA) ligated with *WT1* variant D were constructed as previously described [18]. FaDu cells were transiently transfected with 3 μg *WT1D* pcDNA 3.1 (+) vectors per well in six-well plates (5 × 10^5^ cells/well) using lipofectamine 2000 (Invitrogen).

### MTT assay

Vybrant MTT Cell Proliferation Assay Kit (Invitrogen) was applied to measure cell proliferation. FaDu cells were collected at 0, 24 and 48 hours after transfection and labeled with MTT solution (3-(4.5-dimethyldiazol-2yl)-2.5-diphenyltetrazolium bromide) mixed with SDS-HCL. Absorbance was measured on spectrometer at 570 nm wavelength.

### Western blot

Total protein was extracted using lysis buffer (0.5% NP-40, 0.5% NA-DOC, 0.1% SDS, 150nM NaCl, 50 mM Tris pH 7.5, 1 mM EDTA, 1 mM NaF) supplemented with protease inhibitor (Sigma-Aldrich, St. Louis, MO, USA). Protein concentration was measured using BCA reagent (Thermo Scientific, Rockford, IL, USA). Twenty μg of each sample was separated using 10% SDS polyacrylamide gel electrophoresis (BIO-Rad, Hercules, CA, USA) and then transferred to a PVDF membrane (Millipore, Billerica, MA, USA). The membrane was blocked using TBST containing 5% non-fat dry milk, then incubated with mouse-monoclonal antibodies against WT1 (1:250, catalog no. M3561, DAKO, Glostrup, Denmark), p63 (1:2000, catalog no. M7247, DAKO), p53 (1:1000, catalog no. PAb 1801, Abcam, Cambridge, UK) and β-actin (1:10000, catalog no. MAB1501R, Millipore) followed by a second incubation with peroxidase conjugated anti-mouse polyclonal antibodies (1:5000, DAKO). The antibody (anti-p63) used in this study is able to detect bands corresponding to the expected molecular weights and according to expression patterns of the various isoforms (TAp63α, TAp63γ, ΔNp63α, and ΔNp63γ). Proteins were visualized using a chemiluminescent detection system (ECL-advanced, GE healthcare UK) in ChemiDoc XRS (Bio-Rad, Italy).

### RNA extraction and cDNA preparation

Total RNA was extracted using TRIzol reagent (Invitrogen, Stockholm, Sweden). cDNA was prepared using superscript II reverse transcriptase kit according to the manufacturer’s instructions (Invitrogen).

### Chromatin immunoprecipitation (ChIP)/PCR analysis

ChIP analysis was performed using the Chromatin Immunoprecipitation Kit (Upstate Millipore, Billerica, MA, USA). SKOV-3 cell line, derived from the ascitic fluid of a female with an ovarian tumor (ATCC HTB-77) with no endogenous WT1 expression and null p53 expression (p53 mutation at codon 89 and 179) was used as an extra negative control [19,20]. Approximately 1 × 10^6^ FaDu cells with or without WT1D transfection and SKOV-3 cells were crosslinked with 1% formaldehyde, followed by glycine to quench unreacted formaldehyde. Chromatin was sonicated on ice to shear crosslinked DNA to about 200–1000 bp in length using a sonifier ultrasonic cell disrupter (Branson, Danbury, CT, USA) with 12 × 10s pulses. The sheared chromatin was resuspended in dilution buffer and 1% of the chromatin was removed as input, followed by immunoprecipitation using protein G magnetic beads with 2 μg of either anti-WT1 (C-19) antibody (catalog no. sc-192, Santa Cruz Biotechnology Inc, Santa Cruz, CA, USA) or normal rabbit IgG (catalog no. 2729S, Cell Signalling technology Inc, Danvers, MA, USA) at 4°C overnight with rotation. After the reversal of crosslinks by incubation in ChIP elution buffer containing proteinase K at 62°C for 2 h, DNA was purified using spin columns. PCR reactions containing 2 μl of the immunoprecipitated DNA or input chromatin, primers and AmpliTaq Gold (Applied Biosystem) in a 25 μl volume were performed with initial denaturation at 95°C for 10 min, followed by 35 cycles (95°C for 30 s, 60°C for 30s and 72°C for 45 s) and a final extension at 72°C for 10 min. Primer sequences for *p63* promoters are shown in Additional file 1: Table S1. PCR products were fractioned on 1% agarose gel and ethidium bromide stained DNA was visualized on Ultraviolet Transilluminator (Spectroline, Westbury, NY, USA). For quantitative real-time PCR, SYBR green master mix (Bio-Rad) was used in a 25 μl volume of reaction. For PCR amplification of cDNA, IQ Sybr Green supermix (Bio-Rad) was used, and samples were analyzed on Iq5 (Bio-Rad). The primer sequences are the same as the sequences listed in Additional file 1: Table S1.

### Genome-wide gene expression array

From each sample, 200 ng RNA was used to produce biotinylated cRNA using TargetAmp-Nano labeling kit (Illumina, San Diego, CA, USA). A total of 750 ng biotinylated cRNA was hybridized to an Illumina HumanHT-12 v4 Expression BeadChip according to the manufacturers’ protocol (Illumina). Arrays were scanned using Illumina iScan Reader. The GenomeStudio (Illumina) software was used for data processing. For normalization, background correction and variance stabilization transformation Lumi package was used [21]. Differentially expressed genes were identified based on a moderated *t* test using MEV software package from TIGR [22]. Network analysis was carried out with the Metacore software (GeneGo Inc, St Joseph, MI, USA). Pathway analysis was carried out using the Database for Annotation, Visualization, and Integrated Discovery (DAVID) tool [23].

### Protein co-immunoprecipitation (co-IP)

FaDu cells were lysed in cold lysis buffer (0.5% NP-40, 0.5% NA-DOC, 0.1% SDS, 150nM NaCl, 50 mM Tris pH 7.5, 1 mM EDTA, 1 mM NaF) supplemented with protease inhibitor (Sigma-Aldrich, St. Louis, USA) for 30 min at 4°C; lysates were clarified by centrifugation at 14,000 rpm for 30 min at 4°C. Equivalent amounts of protein lysate were incubated with the anti-WT1 (catalog no. M3561, DAKO, Glostrup, Denmark), anti-IgG (catalog no. 2729S, Millipore, Billerica, U.S.A.) antibodies at 4°C overnight, then incubated with Protein G Sepharose 4 Fast Flow (GE Healthcare, Uppsala, Sweden) at 4°C for 1 hr. Immunoprecipitates were washed with lysis buffer three times. Immunoprecipitated proteins were eluted with SDS-sample buffer and analyzed by SDS-PAGE and Western blotting. Immuno-blotting was conducted using anti-WT1 (1:250, catalog no. M3561, DAKO, Glostrup, Denmark), p53 (1:2000, catalog no. PAb 1801, Abcam, Cambridge, UK) and p63 (1:2000, catalog no. M7247, DAKO, Glostrup, Denmark).

### Patient samples and real-time quantitative PCR

After obtaining informed written consent, tumor biopsies were taken from 15 patients with SCCHN, clinically adjacent tumor-free tissue was available from 7 of the patients. Punch biopsies were taken from 14 healthy non-smoking volunteers. The tissue specimen collection had been approved by the Ethics Committee at Umeå University (Dnr 01–057). *WT1* mRNA level was quantified by real-time quantitative PCR (RT-qPCR) using TaqMan technology in 7900HT system (Applied Biosystems, Foster City, CA, USA). RT-qPCR reactions were carried out in a 25 μL volume containing 12.5 μL universal PCR master mix, each primer at a concentration of 0.5 mM, probe at 0.1 mM, and 50 ng of cDNA. Triplicate assays were run in parallel for each sample. *WT1* transcription values were normalized against the expression of β*-actin*, to adjust for variations in RNA and cDNA synthesis. The mean of triplicates of the *WT1* gene copy numbers was divided by the mean of duplicates of copy numbers of the *β-actin*. Primers and probes for the *WT1* and *β-actin* gene and the amplification conditions have been described previously [24].

### Statistical analysis

Statistical analysis was performed using SPSS (version 19, SPSS Inc., Chicago, IL, USA). Mann–Whitney *U*-test was used to compare differences in the expression of two different variables. Fisher’s exact tests (when sample size was <5) were used for comparison of proportions. A *p-*value < 0.05 was considered to be significant.

## Results

### Altered cell proliferation through knockdown of WT1, p63 and p53

**Figure 1:**
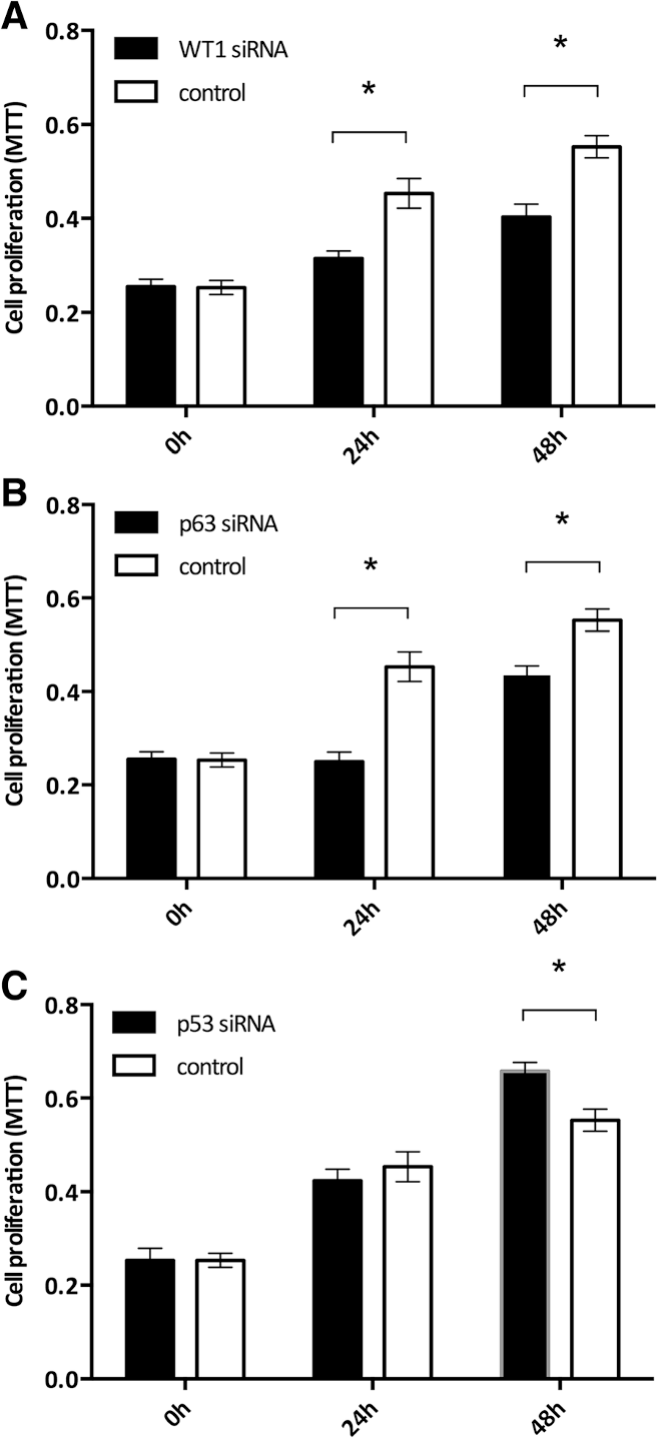
Alterations in cell proliferation by knockdown of WT1, p63 or p53 in FaDu cells. MTT analysis of FaDu cells transiently transfected with siRNA targeting *WT1***(A)**, *p63***(B)** and *p53***(C)**. *p < 0.05.

To determine the effect of WT1, p63 and p53 on cell proliferation in FaDu cells *in vitro*, MTT assays were performed. Knockdown of *WT1* resulted in a significant decrease in cell proliferation at 24 and 48 hours after transfection (*p* < 0.05, Figure 1A). Similarly, silencing *p63* RNA induced a considerable decrease in cell proliferation at both time points (*p* < 0.05, Figure 1B). These results indicate that both WT1 and p63 have a positive effect on cell proliferation in FaDu cells.

p53 function is inactivated in up to 80% of HNSCC [25]. In the FaDu cell line, *p53* has a point mutation at codon 248 (Arg → Leu) [26]. The R248L mutation of *p53* does not completely abolish its inhibitory effect on cell proliferation in this cell line. As shown in Figure 1C, a significant increase in cell proliferation in p53 knockdown cells was demonstrated at 48 hours after transfection compared to control cells (*p* < 0.05).

### Correlation between WT1 expression and p63/p53 in FaDu cells

Previous studies have demonstrated a protein-protein interaction between WT1 and p63/p53 [16,27]. Furthermore, WT1 has been reported to exert protein stabilization on p53 in some cellular settings [15]. In order to study the relationship between WT1 and p63/p53 in SCCHN, transfection experiments in FaDu cells were performed. Suppressed expression of WT1, p63 and p53 were induced using siRNA technologies.

**Figure 2:**
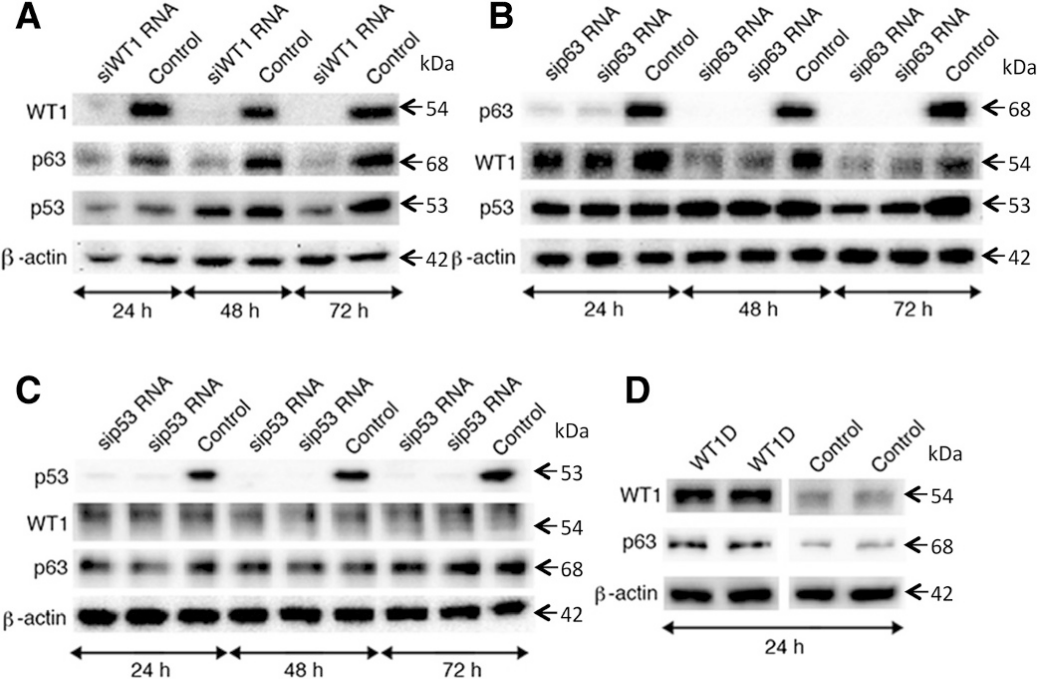
Alterations of protein expression of WT1 and p63/p53 using *in vitro* experiments in FaDu cells, demonstrated by western blot. Cells were harvested at 24, 48 or 72 hours after transient transfection with siRNA targeting *WT1***(A)***p63***(B)***p53***(C)** and after *WT1D* plasmid transfection at 24 hours **(D)**.

Successful silencing of *WT1* RNA resulted in downregulated expression of WT1 protein as seen on western blot (Figure 2A). Distinctly decreased expression of ΔNp63 (68 kDa) was observed in cells with suppressed WT1 expression compared to control cells. However, we found that expression of the TAp63α (75 kDa) was much weaker than ΔNp63α (68 kDa). TAp63α showed no changes in expression in our experiments. γ-isoforms (TAp63γ or ΔNp63γ) were not detectable in FaDu cells (data not shown). A slight decrease in protein expression of p53 in WT1 knockdown cells was observed only at 72 hours after transfection.

Knockdown of p63 induced a slight decrease in protein expression of WT1 at 48 and 72 hours after transfection (Figure 2B). Decreased expression of p53 was observed only at 72 hours after transfection.

No alterations of WT1 or p63 protein expression were observed in p53 knockdown cells (Figure 2C).

An additional experiment was performed to confirm the positive correlation between WT1 and ΔNp63 using a plasmid carrying *WT1D* variant into FaDu cells. Upregulation of ΔNp63 protein levels was observed in cells with forced overexpression of WT1D (Figure 2D). Again, altered expression of TAp63α was not found (data not shown).

These results indicate a possible functional link between WT1 and p63 in FaDu cells, but not a strong association between WT1 and p53 expression.

### *p63* is a WT1 target gene

**Figure 3:**
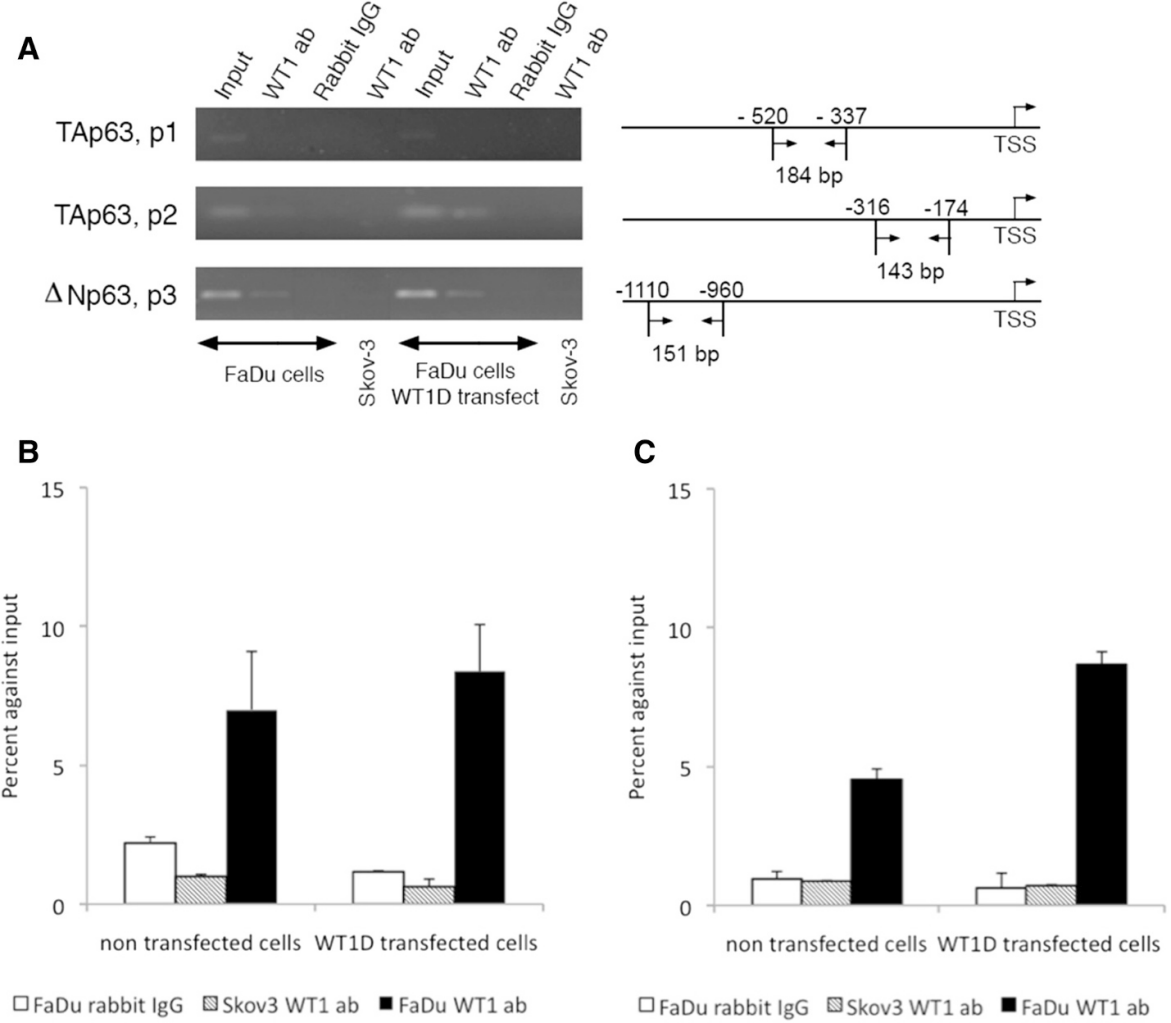
WT1 binds to the promoters of the *p63* gene. ChIP/PCR analysis of *WT1D* transfected and non-transfected FaDu cells. **A**. PCR analysis of the precipitate using p1, p2 and p3 primer pairs. Size and location of the amplified products are depicted on the right. **B** and **C**. RT-qPCR analysis of the precipitate using the p2 **(B)** and p3 **(C)** primer pairs.

A positive correlation between *WT1* and *p63* gene expression was found as described above. To assess whether *p63* is a target gene of WT1, the binding properties of WT1 to the *p63* promoters were examined using ChIP/PCR. Two putative GNGNGGGNG WT1-binding sites in the *TAp63* promoter and one putative WT1-binding site in the Δ*Np63* promoter were identified by sequencing analysis (Additional file 1: Table S1). ChIP was performed with *WT1D* transfected and non-transfected FaDu cells and chromatin precipitated with WT1 antibodies. PCR amplification products could be demonstrated in the region of the second WT1-binding site of the *TAp63* promoter and at the *ΔNp63* WT1-binding site (Figure 3A). Results were also confirmed with quantitative real-time PCR (Figure 3B for the *TAp63* second binding site and Figure 3C for the *ΔNp63* WT1-binding site). Consequently, using ChIP/PCR assay we could demonstrate direct binding of WT1 to the *p63* promoters.

### WT1 can regulate p63 transcription through multiple genes involved in cell growth

**Figure 4:**
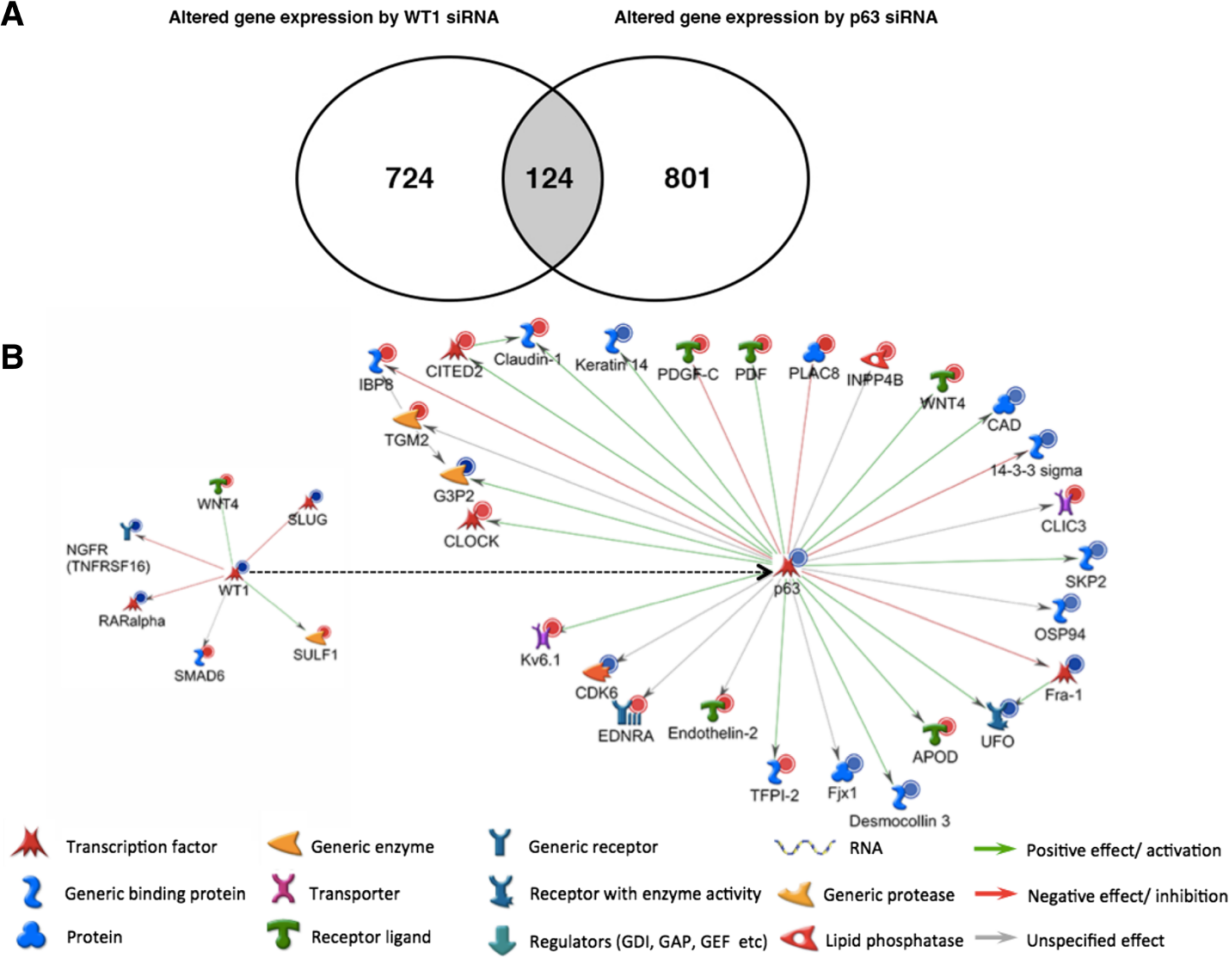
WT1 regulates p63 transcription through multiple genes with microarray analysis. **A**. Venn diagram of the number of differentially expressed genes with a fold change greater than two and a *p* value less than 0.05 following *WT1* or *p63* gene knockdown in FaDu cells. WT1 and p63 regulated genes displayed an overlap of 124 genes. **B**. Altered gene expression of known WT1 and p63 target genes by WT1 siRNA transfection in FaDu cells. Network analysis was performed based on array data using GeneGo software. Increased gene expression is indicated by a red circle on the upper right corner of each network object, whereas a blue dot indicates downregulation. Different shapes and colors represent various gene/protein function.

Genes with altered expression in response to knockdown of WT1 or p63 were detected with microarray analysis. Silencing *WT1* RNA induced significant fold changes of 848 genes compared to control (Figure 4A). Significantly altered expression of 925 genes was found in cells with suppressed p63 expression. Interestingly, by combining the two profiles we found that 124 genes had significantly altered fold changes (*p* < 0.05, Figure 4A). Eighteen of these genes were found to be involved in cell proliferation, cell cycle regulation and DNA replication (Table 1). Ten genes involved in cell proliferation, five genes involved in cell cycle regulation and three genes associated with DNA replication were significantly altered in WT1 and p63 knockdown cells (*p* < 0.005, Table 1).

**Table 1:** **Significant fold changes of expression of genes involved in cell proliferation, cell cycle regulation and DNA replication by knockdown of WT1 or p63 in FaDu cells**

Term	Gene name	Expected effect*	Fold change (vs control)	
			siWT1 RNA	sip63 RNA
**Cell proliferation**	MMP7	Activator	2.11	4.11
	NGFR	Activator	0.47	0.36
	IL8	Activator	0.46	2.22
	IGFBP3	Suppressor	2.63	2.85
	RARRES1	Suppressor	2.48	8.36
	TIMP2	Suppressor	2.12	2.07
	CDKN1B	Suppressor	2.09	2.45
	LDOC1	Suppressor	2.01	2.69
	TOB2	Suppressor	0.48	0.36
	SFN	Suppressor	0.41	0.38
**Cell cycle**	TGM2	Activator	4.14	4.22
	Skp2	Activator	0.49	0.46
	C13orf15	Activator/Suppressor	4.07	15.17
	SMAD6	Unspecified	3.19	3.06
	CITED2	Unspecified	2.30	3.18
**DNA replication**	MCM3	Activator	0.48	0.49
	MCM5	Activator	0.40	0.48
	RFC3	Activator	0.37	0.44

*Expected effect of the listed genes was based on previous studies.

Five negative regulators of cell proliferation *IGFBP3*, *RARRES1*, *TIMP2*, *CDKN1B*, *LDOC1* and one positive regulator, *MMP7*, demonstrated increased expression. Two suppressors, *TOB2* and *SFN* and one activator, *NGFR*, showed decreased expression. *TGM2*, a positive regulator of cell cycle progression and *C13orf15*, which has been described as both activator and suppressor of cell cycle progression, demonstrated increased expression. *Skp2*, another activator of cell cycle progression showed decreased expression. All three positive regulators of DNA replication, *MCM3, MCM5* and *RFC3* demonstrated decreased expression. Interestingly, *IL8*, an activator of cell proliferation, demonstrated decreased expression in *WT1* knockdown cells, but increased expression in p63 knockdown cells. No genes associated with apoptosis were found to be altered in the combined profiles. However, knockdown of p63 was found to induce alterations in the transcription of 24 genes involved in apoptosis.

In addition, by using Metacore GeneGo analysis, 6 known WT1 target genes and 27 known p63 downstream target genes were found to be affected in WT1 knockdown cells (Figure 4B). In p63 knockdown cells, 44 known p63 target genes were affected (Additional file 2: Figure S1). Among those p63 target genes, ten demonstrated altered expression in both WT1 knockdown and p63 knockdown cells (Table 2). Expression of four genes was significantly decreased by p63 and WT1 siRNA transfection. *SFN* is known to be repressed by p63 while *Skp2* and *CAD* can be activated by p63. In contrast, significantly increased expression of six genes was shown (Table 2). *CITED2* and *GDF2* are known to be activated by p63 whereas *PLAC8* and *IGFBP3* are repressed by p63. The effects of p63 on *Fjx1*, *INPP4B* and *TGM2* are unspecified. Taken together, these genes are known to be involved in cell cycle, cell growth, cell migration, cell proliferation, inositol phosphate metabolism and pyrimidine metabolism.

**Table 2:** **Fold changes in expression of known p63 target genes in response to WT1 and p63 gene knockdown in FaDu cells**

Gene name	Expected effect by p63*	Fold change (vs control)		Gene function
		siWT1	sip63	
**SFN**	Repressed	0.41	0.38	Cell proliferation
**Skp2**	Activated	0.49	0.46	Cell cycle
**CAD**	Activated	0.50	0.47	Pyrimidine metabolism
**Fjx1**	Unspecified	0.44	0.47	Cell growth
**INPP4B**	Unspecified	1.10	1.27	Inositol phosphate metabolism
**CITED2**	Activated	1.20	1.67	Cell cycle
**TGM2**	Unspecified	2.05	2.08	Cell cycle
**PLAC8**	Repressed	2.51	2.43	Cell migration
**GDF15**	Activated	1.18	2.66	Cell migration
**IGFBP3**	Repressed	2.63	2.85	Cell proliferation

*Expected effect of the listed genes was based on previous studies.

### WT1 protein interacts with p53 but not p63

**Figure 5:**
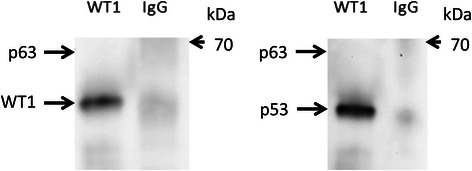
Protein interactions between WT1 and p53 but not p63 by co-IP. Equivalent amounts of protein lysate from FaDu cells were incubated with the anti-WT1, anti-IgG antibodies, followed by incubation with Protein G Sepharose 4 Fast Flow. Immunoprecipitated proteins were analyzed by Western blotting. Immuno-blotting was conducted using anti-WT1, p53 and p63.

In order to study the protein interaction between WT1 and p53/p63, co-IP analysis was performed. As shown in Figure 5, p53 was detected in WT1 immune-complexes but not p63, indicating protein interaction occurred between *WT1* and *p53* in FaDu cells.

### High *WT1* RNA expression in clinical samples

*WT1* RNA expression levels were analyzed by real-time quantitative PCR (RT-qPCR) in 15 SCCHN tumor specimens, 7 adjacent tumor-free tissue samples and 14 normal control tissues of the tongue. Significantly higher *WT1* mRNA levels were detected in tumor specimens compared to adjacent tumor-free tissue samples (Additional file 3: Figure S2, p < 0.001) and normal control tongue tissues (Additional file 3: Figure S2, p=0.001), indicating overexpression of *WT1* in SCCHN. No significant correlation was found between *WT1* mRNA levels and clinical features including age, sex, tumor stage, overall survival and disease specific survival (data not shown). Using immunohistochemistry, we performed WT1 protein staining in 90 formalin-fixed tumour samples and found that only 5 out of 90 samples showed positive staining in cytoplasm.

## Discussion

In the present study we found a novel positive correlation between *WT1* and *p63* gene expression and further confirmed that WT1 regulates *p63* expression through direct binding to the *p63* promoters. Both WT1 and p63 were found to promote cell proliferation in SCCHN cells. Further, *in vitro* experiments showed altered expression of 18 genes involved in cell proliferation, cell cycle regulation and DNA replication shared by silencing of *WT1* and *p63* RNA. Several known WT1 and p63 target genes were affected by knockdown of WT1. Additionally, *WT1* mRNA levels were overexpressed in SCCHN samples.

Using *in vitro* experiments, we found decreased cell proliferation due to loss of *WT1* in FaDu cells. WT1 isoform D was recently found to induce cell proliferation in oral squamous cell carcinoma cells, a subtype of SCCHN [28]. Furthermore, increased cell proliferation induced by WT1 has been shown in several other types of cancer cells including non-small cell lung cancer [11] and several solid cancer cells [29]. The collected data suggest that *WT1* functions as an oncogene in these neoplasms.

Overexpression of *p63* has been found in a majority of patients with squamous cell carcinomas and SCCHN [30]. In FaDu cells, ∆Np63 has been found to be the main isoform [6,31]. One previous study has shown that knockdown of the ∆Np63 isoform, but not the TAp63 isoform inhibits cell proliferation in some SCCHN cell lines [32]. However, another study has shown that the silencing of ∆Np63 in FaDu cells does not alter the proliferation state, as judged by Ki-67 expression and FACS analysis regarding cell cycle phase DNA content [4]. In the present study decreased cell proliferation was observed in p63 knockdown cells, showing that p63 can promote cell proliferation in FaDu cells and overexpression of ∆Np63 isoform was detected by western blot. Our results support the expected oncogenic role of the *p63* gene in this cell line.

The FaDu cell line contains a point mutation of *p53* at codon 248 (Arg → Leu) [26], one of the most frequent mutation sites of the gene [25]. Codon 248 is located in the DNA binding domain and mutations in this specific location has suggested generating a protein incapable of binding to target DNA, thereby losing its regulatory function on transcription [33]. Failure of induction of p53-dependent apoptosis has previously been demonstrated in FaDu cells [34]. However, we observed that p53 had an inhibitory effect on cell proliferation. The same mutation in H322a, a non-small cell lung cancer cell line, showed that mutant p53^R248L^ still possesses a tumor suppressor function, as demonstrated by expansion of cell proliferation due to reduction in gene expression [35].

WT1 is known to regulate transcription of an extensive number of genes [36]. In this study we found a strong positive correlation between WT1 and p63 and confirmed that the WT1 protein binds to the *p63* promoters, assessed by ChIP/PCR analysis which showed that p63 is a target gene of WT1. A direct binding of WT1 protein to the promoters of the two main p63 isoforms, TAp63 and ∆Np63 was demonstrated. However, the WT1 binding site (P1,-502 to --493), far from the major transcription start site in the *TAp63* promoter, was not involved. We did not find any altered TAp63 expression in our experiment. Low efficiency may be explained by very low expression of TAp63 in FaDu cells by western blot and only one binding site on TAp63 promoter by WT1 protein by ChIP/PCR. As mentioned previously, ∆Np63 is the only major isoform expressed and the isoform that plays a major functional role in FaDu cells.

Previous studies have presented evidence for a protein-protein interaction between WT1 and p53 in baby rat kidney [37] cells, as well as in Wilms’ tumors [27]. A *p53* mutation at position homologues to human codon 248 in BRK cells did not abolish this interaction. Furthermore, WT1-induced p53 protein stabilization has been reported in Saos-2 cells [15]. In this study, we also showed that WT1 interact with p53 in FaDu cell by using Co-IP analysis and observed decreased protein levels of p53 in cells with suppressed WT1 expression at 72 hours. Results may be explained by previous findings regarding p53 protein stabilization. In contrast to previous study [16], protein interaction between WT1 and p63 was not detected in FaDu cells.

Microarray analysis showed that 18 genes involved in cell proliferation, cell cycle regulation and DNA replication were significantly altered in both WT1 and p63 knockdown cells. Five of these genes were previously described as p63 target genes. ∆Np63 has been reported to directly repress the expression of the p53-target genes *IGFBP-3* [38] and *SFN* (*14-3-3σ*) [39], supporting the known dominant negative effect of ∆Np63 regarding p53 function [5]. *CITED2* and *Skp2* were also previously identified target genes of p63 [40,41]. *CDKN1B* (*p27*^*kip1*^) expression has been shown to be inversely correlated to *∆Np63* expression, suggesting a possible direct negative regulation of ∆Np63 on *CDKN1B* transcription [32]. The fold changes of 11 of these 18 genes were almost identical. An indirect regulation of p63 target genes as major mechanism for WT1 regulation of listed genes is therefore not likely. According to immunoblot results, WT1-knockdown cells express p63 at a reduced level, still enabling transcriptional regulation as opposed to p63-knockdown cells*. MMP7*, *RARRES1*, *C13orf15* and *CITED2* are genes showing a distinct difference between WT1 and p63 knockdown cells. These genes were all shown to be repressed by both p63 and WT1, but to a greater extent by p63. Indirect regulation by WT1 might serve as regulation of those genes. *CITED2*, as mentioned previously is the only known p63 target gene of the above listed genes [40].

MMP-7 is a matrix degrading protein usually associated with tumor invasion and angiogenesis in cancer progression [42], but has also been linked to induction of proliferation [43] and apoptosis [44]. In contrast to these findings, we showed increased fold changes of *MMP7* expression in both WT1 and p63 knockdown cells. *MMP-7* has been reported to be overexpressed in SCCHN [45].

Previous studies have shown contradictory functions for the *RGC-32* gene (C13orf15). RGC-32 has been reported to promote cell cycle progression and thereby cell proliferation [46]. However, tumor suppressor properties of the *RGC-32* gene have also been reported. *RGC-32* has been identified as a p53 target gene with an ability to inhibit cell proliferation by the induction of G2/M arrest [47]. *RGC-32* was found in the present study to be extensively upregulated in p63 knockdown cells. Our results suggest that *RCG-32* may act as a tumor suppressor in FaDu cells.

Interestingly, *IL8* demonstrated decreased expression when silencing WT1, but an increased fold change when knocking down p63. IL8 is known to be a pro-inflammatory chemokine that responds to the activation of NF-κβ. IL8 induces angiogenesis through activation of endothelial cells and has been reported to act as an autocrine growth factor inducing cell proliferation [48]. A recent study showed that ∆Np63 can bind to the *IL8* promoter and alter gene expression when interacting with RelA or cRel, members of the NF-κβ family [49]. Contrary to the observations in our *in vitro* experiment, ∆Np63 has previously shown to have an activating effect on *IL8* transcription in SCCHN cells [50]. Association between WT1 and *IL8* expression has not previously been reported. Further studies are therefore needed to investigate whether WT1 regulates *IL8* expression directly or indirectly.

The effects of WT1 and p63 on cell proliferation observed in this study can be explained by their regulation of many genes involved in proliferation, cell cycle processes and DNA replication. Additionally, WT1 was found to regulate genes involved in the p53, Wnt and PI3K/AKT-1 signaling pathways, giving further ground for the proliferative effect of WT1 in FaDu cells. In the present study we suggested that WT1 could inhibit the p53-signaling pathway through transcriptional regulation of activators and repressors of the pathway. No alterations of apoptosis-regulating genes were found in WT1-depleted cells, suggesting a possible alteration of this signaling pathway through cell cycle arrest and transcriptional activation of DNA repair genes. Furthermore, in this study we could not detect any pattern of up- or downregulation of the Wnt or PI3K/AKT-1 pathways. However, earlier studies have identified nine genes in the Wnt signaling pathway to be direct targets of WT1 [51]. The PI3K/AKT-1 pathway has been implicated in WT1 signaling in lung cancer [52].

Using Metacore GeneGo software we found that expressions of ten known p63 target genes were altered in both WT1 and p63 knockdown cells. These genes were involved in the cell cycle, cell growth, cell migration, cell proliferation, inositol phosphate metabolism and pyrimidine metabolism. *SFN* was previously found to be negatively regulated by ΔNp63 in primary human epidermal keratinocytes (HEKs) as described above [39]. *Skp2* expression has been found positively regulated by p63 in HEKs [41]. Using ChIP-on-chip array analysis, Huang et al. found that the ΔNp63 protein could bind to the *CAD* promoter in squamous cell carcinoma cells when cells were exposed to cisplatin [53]. A previous study showed that p63 could activate the *CITED2* promoter in keratinocytes [54]. In human keratinocytes, HaCaT, TAp63 was found to activate *GDF15* by directly binding to the promoter [55]. The proapoptotic protein IGFBP-3 has been shown to be negatively regulated by ΔNp63α in the squamous epithelial cell lines HaCaT and SCC-1 [38]. However, these known p63 target genes have not been reported correlated with WT1. Further studies are needed to find out whether WT1 can directly regulate these genes.

In agreement with a study by Oji et al. [17], overexpression of *WT1* was detected in SCCHN tissue samples in our patient cohort. In a study by Mikami et al., WT1 mRNA was found to be overexpressed in one of six cell lines from oral squamous cell carcinoma. Immunohistochemical analysis of tissue sections showed overexpression of WT1 protein in two of 29 patients with oral squamous cell carcinoma, suggesting that WT1 plays an important role in the pathogenesis of some types of oral squamous cell carcinoma [56]. No correlation between *WT1* mRNA levels and clinical parameters such as age, sex, tumor stage and overall survival was observed in our limited patient cohort. The potential prognostic impact should, however, be studied in larger patient cohorts.

## Conclusions

Our experimental results in FaDu cells indicate oncogenic roles for *WT1* and *p63* in SCCHN cells. We reported for the first time that WT1 can directly regulate *p63* expression and induce an effect on several known p63 target genes. Therefore, therapeutic approaches targeting the WT1 and p63 proteins might serve as alternative treatment in SCCHN. These findings may warrant further investigation regarding the effects of WT1 and p63 inhibitors *in vitro* and *in vivo*.

## Additional files

Additional file 1: Table S1. Primers used for amplification of *p63* promoters regions.
Additional file 2: Figure S1. Altered gene expression of known p63 target genes was found by p63 siRNA transfection in FaDu cells. Network analysis was performed based on array data using GeneGo software. Increased gene expression is indicated by a red circle on the upper right corner of each network object, whereas a blue dot indicates downregulation. Different shapes and colors represent various gene/protein functions.
Additional file 3: Figure S2. *WT1* mRNA levels in tongue tumor tissue samples compared to adjacent tumor-free tissues or normal control tongue tissue.

## Abbreviations

WT1: Wilms’ tumor gene 1
SCCHN: Squamous cell carcinoma of the head and neck
siRNA: Silencing RNA
ChIP: Chromatin immunoprecipitation
Co-IP: Co-immunoprecipitation
DAVID: Database for annotation, visualization, and integrated discovery
RT-qPCR: Real-time quantitative PCR
BRK: Baby rat kidney
HEKs: Human epidermal keratinocytes
