# Supplementary figures and images for: Wilms’ tumor gene 1 regulates p63 and promotes cell proliferation in squamous cell carcinoma of the head and neck
Source: BMC Cancer. 2015 May 1;15:342. doi: 10.1186/s12885-015-1356-0 (PMC4421988; doi:10.1186/s12885-015-1356-0)

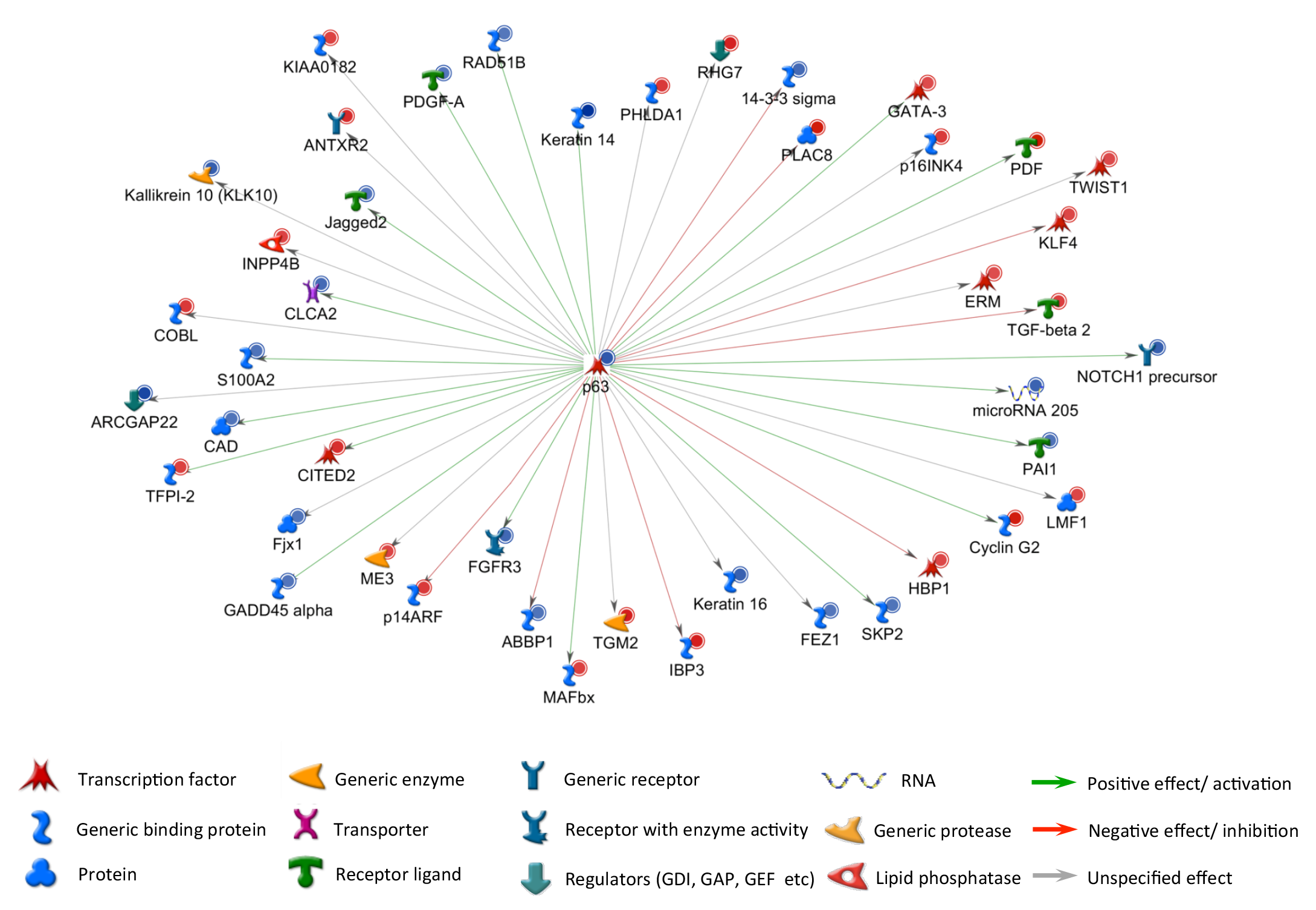

Supplement: Additional file 2: Figure S1. — Altered gene expression of known p63 target genes was found by p63 siRNA transfection in FaDu cells. Network analysis was performed based on array data using GeneGo software. Increased gene expression is indicated by a red circle on the upper right corner of each network object, whereas a blue dot indicates downregulation. Different shapes and colors represent various gene/protein functions. [file 12885_2015_1356_MOESM2_ESM.tiff]

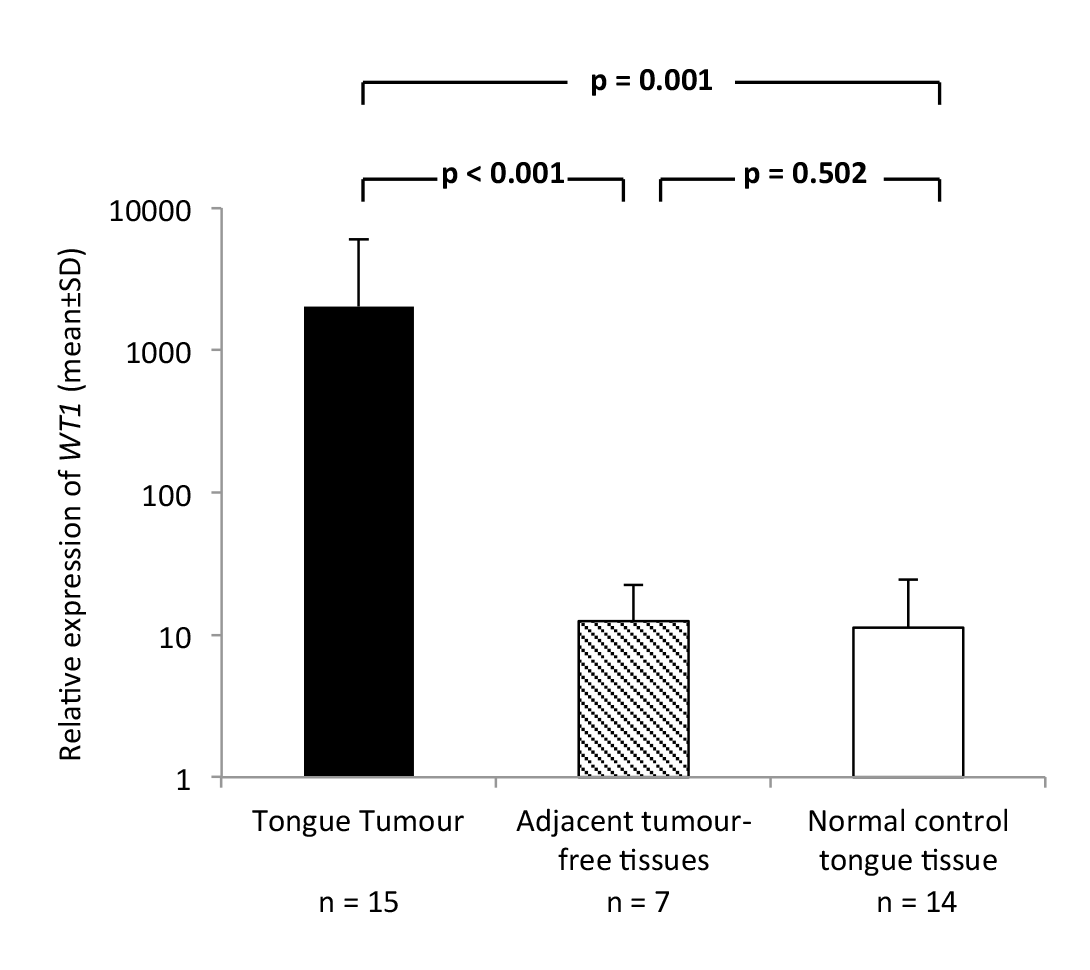

Supplement: Additional file 3: Figure S2. — WT1 mRNA levels in tongue tumor tissue samples compared to adjacent tumor-free tissues or normal control tongue tissue. [file 12885_2015_1356_MOESM3_ESM.tiff]
